# A recurrent and transesophageal echocardiography–associated outbreak of extended-spectrum β-lactamase–producing *Enterobacter cloacae* complex in cardiac surgery patients

**DOI:** 10.1186/s13756-019-0605-4

**Published:** 2019-09-18

**Authors:** Tom Van Maerken, Els De Brabandere, Audrey Noël, Liselotte Coorevits, Pascal De Waegemaeker, Raina Ablorh, Stefaan Bouchez, Ingrid Herck, Harlinde Peperstraete, Pierre Bogaerts, Bruno Verhasselt, Youri Glupczynski, Jerina Boelens, Isabel Leroux-Roels

**Affiliations:** 1 Department of Biomolecular Medicine, Ghent University, Ghent, Belgium; 2 Department of Laboratory Medicine, AZ Groeninge, Kortrijk, Belgium; 3 Department of Infection Control, Ghent University Hospital, Ghent, Belgium; 4 Laboratory of Clinical Microbiology, Belgian National Reference Center for Monitoring Antimicrobial Resistance in Gram-Negative Bacteria, CHU UCL Namur, Yvoir, Belgium; 5 Department of Laboratory Medicine, Ghent University Hospital, Ghent, Belgium; 6 Department of Diagnostic Sciences, Ghent University, Ghent, Belgium; 7 Department of Anesthesiology, Ghent University Hospital, Ghent, Belgium; 8 Department of Intensive Care Medicine, Ghent University Hospital, Ghent, Belgium

**Keywords:** Outbreak, Cardiac surgery, *Enterobacter cloacae* complex, Extended-spectrum β-lactamase, Transesophageal echocardiography

## Abstract

**Background:**

We report a recurrent outbreak of postoperative infections with extended-spectrum β-lactamase (ESBL)–producing *E. cloacae* complex in cardiac surgery patients, describe the outbreak investigation and highlight the infection control measures.

**Methods:**

Cases were defined as cardiac surgery patients in Ghent University Hospital who were not known preoperatively to carry ESBL-producing *E. cloacae* complex and who postoperatively had a positive culture for this multiresistant organism between May 2017 and January 2018. An epidemiological investigation, including a case-control study, and environmental investigation were conducted to identify the source of the outbreak. Clonal relatedness of ESBL-producing *E. cloacae* complex isolates collected from case patients was assessed using whole-genome sequencing–based studies.

**Results:**

Three separate outbreak episodes occurred over the course of 9 months. A total of 8, 4 and 6 patients met the case definition, respectively. All but one patients developed a clinical infection with ESBL-producing *E. cloacae* complex, most typically postoperative pneumonia. Overall mortality was 22% (4/18). Environmental cultures were negative, but epidemiological investigation pointed to transesophageal echocardiography (TEE) as the outbreak source. Of note, four TEE probes showed a similar pattern of damage, which very likely impeded adequate disinfection. The first and second outbreak episode were caused by the same clone, whereas a different strain was responsible for the third episode.

**Conclusions:**

Health professionals caring for cardiac surgery patients and infection control specialists should be aware of TEE as possible infection source. Caution must be exercised to prevent and detect damage of TEE probes.

## Background

Members of the *Enterobacter cloacae* complex are part of the commensal intestinal flora in humans and can act as opportunistic pathogens to cause serious nosocomial infections. These facultative anaerobic Gram-negative bacilli are intrinsically resistant to ampicillin, amoxicillin, amoxicillin–clavulanic acid, first-generation cephalosporins and cefoxitin due to the production of constitutive AmpC β-lactamase [1]. Plasmid-mediated β-lactam resistance is increasingly reported in *E. cloacae* complex strains worldwide. The most recent estimates in Belgian acute care hospitals indicate that 12.4 and 1.6% of all *E. cloacae* complex strains are producing extended-spectrum β-lactamases (ESBLs) and carbapenemases, respectively [2].

We have been confronted with an increased incidence of ESBL-producing *E. cloacae* complex infections in cardiac surgery patients in Ghent University Hospital from May 2017 onwards. Epidemiological investigation pointed to transesophageal echocardiography (TEE) as the source of the outbreak. Despite initially successful institution of infection control measures, the outbreak recurred twice over the course of 9 months. We report here the different outbreak episodes and shed light on a number of critical aspects that should be taken into account to prevent and control similar epidemics in other hospitals.

## Methods

### Clinical setting

Ghent University Hospital is a 1062-bed tertiary care hospital in East Flanders, Belgium. Approximately 1000 cardiac operations on adult and pediatric patients are performed annually by six cardiac surgeons in two designated cardiac operating rooms. Patients are admitted postoperatively to a 10-bed cardiac surgery intensive care unit (CSICU), where they are treated until sufficiently recovered to be transferred to a 28-bed cardiac surgery ward. The hospital has an infection control team composed of two physicians, three nurses and three administrative staff members.

### Case definition

Cases were defined as patients who underwent cardiac surgery in Ghent University Hospital between May 2017 and January 2018 and who subsequently developed colonization or infection with ESBL-producing *E. cloacae* complex as demonstrated by a positive culture from any site. Patients known preoperatively to carry ESBL-producing *E. cloacae* complex were excluded from the case definition. Systematic preoperative screening of cardiac surgery patients for multiresistant Gram-negative bacteria was installed end of June 2017, implying that the preoperative microbiological status is not known for all cases in the first 1.5 months of the outbreak. An outbreak episode was defined as at least two cases linked by a temporal and epidemiological chain of transmission.

### Epidemiological investigation

Several outbreak meetings were held between the hospital infection control team and cardiac surgeons, anesthesiologists, intensivists, cardiologists, operating room nurses and intensive care nurses to identify the source of the outbreak and to discuss and implement infection control measures. Medical records of all cases were reviewed to uncover common factors. The care pathway of cardiac surgery patients was evaluated by the infection control team, including assessment of preoperative care, direct observation of cardiac operations and hand hygiene practices, inspection of wound and airway care and other care-related activities at CSICU, assessment of antibiotic prescribing, analysis of reprocessing of equipment and evaluation of cleaning and disinfection procedures. Special emphasis was given to the use and disinfection of TEE probes, as TEE examinations are typically performed in cardiac surgery patients and as most cases presented with ESBL-producing *E. cloacae* complex in respiratory samples. Preoperative screening of rectal and throat swabs and weekly postoperative screening of rectal swabs for multiresistant Gram-negative bacteria were carried out in all cardiac surgery patients after recognition of the outbreak at the end of June 2017.

Baseline incidence, attack rate and post-outbreak incidence of ESBL-producing *E. cloacae* complex at CSICU were quantified in the period from January 2017 to June 2018 by dividing the monthly number of new CSICU patients with an ESBL-producing *E. cloacae* complex isolate from any site, with ‘new’ being defined as not previously known at any ward or intensive care unit (ICU) as being colonized or infected, by one hundredth of the monthly number of admissions at CSICU. Similar incidence rates per 100 admissions were calculated for the cardiac intensive care unit (CICU), the medical intensive care unit (MICU), the non-cardiac surgical intensive care unit (SICU) and the entire hospital excluding CSICU.

A retrospective case-control study was performed on the patients from the first outbreak episode. As the number of cases was limited, two controls were included per case in order to increase statistical power. The selected controls were those two cardiac surgery patients who did not show ESBL-producing *E. cloacae* complex in any of their samples during the entire hospital stay and who had their operation as close as possible to the cardiac surgery of the case patient. The following preoperative, intraoperative and postoperative characteristics were retrieved from the medical records and compared between cases and controls: age, gender, body mass index (BMI), smoking habit, medical history, preoperative length of stay, preoperative ICUs or wards, preoperative medication, preoperative American Society of Anesthesiologists (ASA) score, type of cardiac surgery, emergency setting of the operation, operating room, surgeons, anesthesiologists, surgical antibiotic prophylaxis, sternotomy, extracorporeal circulation (ECC), duration of surgery, duration of ECC, intraoperative TEE, postoperative TEE, need for revision, beds occupied at CSICU, length of stay at CSICU, postoperative length of stay, total length of stay and outcome.

### Environmental investigation

Environmental surveillance samples were collected by the hospital infection control team at several time points during the outbreak. Sampled materials included the different TEE probes used in cardiac surgery patients, keyboards of the ultrasound machines, protective foam covers for the TEE probe tips, a storage case for the TEE probe at CSICU, ultrasound transmission gel, caps of the ultrasound gel tubes, warm water mattresses in the operating rooms, water from extracorporeal membrane oxygenation systems and pillows for neonates at CSICU.

### Microbiological methods

Clinical samples were processed and cultured in the microbiology laboratory using standard diagnostic protocols. Rectal and throat screening swab specimens were grown on selective chromogenic agar plates (chromID ESBL; bioMérieux, Marcy l’Etoile, France). Environmental surveillance samples were cultured using direct plating and enriched broth techniques. Suspicious colonies were identified by matrix-assisted laser desorption/ionization time-of-flight mass spectrometry (MALDI-TOF MS) on a Microflex LT instrument (Bruker Daltonics, Bremen, Germany). Antibiotic susceptibility testing of *E. cloacae* complex isolates was performed on an Adagio automated system (Bio-Rad, Hercules, CA, USA) using the disk diffusion method and interpreted according to European Committee on Antimicrobial Susceptibility Testing (EUCAST) breakpoints. Phenotypic identification of ESBL production was based on double disk synergy testing using cefotaxime, ceftazidime, amoxicillin–clavulanic acid and piperacillin–tazobactam. A combination disk diffusion test using cefepime and cefepime–clavulanic acid was performed to confirm ESBL production during the third outbreak episode.

### Molecular methods

Molecular characterization of ESBL-producing *E. cloacae* complex isolates was performed by whole-genome sequencing. Genomic DNA was extracted from the available outbreak isolates and from three unrelated ESBL-producing *E. cloacae* complex isolates that had been cultured from rectal screening swabs of non-cardiac surgery patients hospitalized at the wards of geriatry, abdominal surgery and pneumology in October and November 2017. DNA libraries were prepared using the Nextera XT DNA Library Preparation Kit (Illumina, San Diego, CA, USA) and sequenced as 300-bp paired-end reads on a MiSeq system (Illumina). Sequencing reads were assembled using CLC Genomics Workbench version 11.0 (Qiagen, Hilden, Germany). Assembled contigs were uploaded to ResFinder version 3.0 to detect the presence of acquired antimicrobial resistance genes [3, 4]. Multilocus sequence typing (MLST) of the isolates was performed by submitting the contigs to the MLST version 1.8 web service [5, 6].

### Statistical analysis

Data of the case-control study were processed and analyzed using SPSS Statistics version 25 (IBM, Armonk, NY, USA). Differences in the distribution of categorical variables between cases and controls were assessed by computing odds ratios (ORs) and corresponding 95% confidence intervals (CIs) and by performing Fisher’s exact tests. A Haldane’s correction, consisting of addition of 0.5 to all four cells of the contingency table, was applied in the OR calculation if unadjusted values resulted in division by zero. Differences in the distribution of continuous variables between cases and controls were analyzed by Mann–Whitney *U* tests. All statistical tests were two-sided and were considered significant at *P* < .05.

## Results

### First outbreak episode

**Fig. 1:**
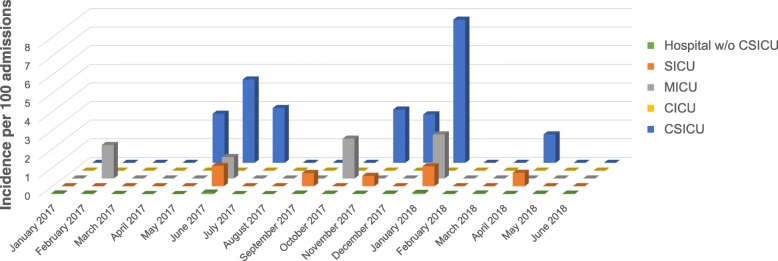
Monthly incidence rates of colonization or infection with ESBL-producing *E. cloacae* complex at CSICU compared to other intensive care units and to the rest of the hospital. Monthly incidence rates are shown per 100 admissions for the period from January 2017 to June 2018. Abbreviations: *CICU* cardiac intensive care unit, *CSICU* cardiac surgery intensive care unit, *ESBL* extended-spectrum β-lactamase, *MICU* medical intensive care unit, *SICU* surgical intensive care unit, *w/o* without

A cluster of postoperative infections with ESBL-producing *E. cloacae* complex in cardiac surgery patients was signalled to the infection control team of Ghent University Hospital in June 2017. Analysis of the monthly incidence rates of patients with a positive culture for ESBL-producing *E. cloacae* complex showed an epidemic increase at CSICU that had started in May 2017 (Fig. 1). Eight cases were detected between May and July 2017. Table 1 shows the demographic and clinical characteristics of the cases, the treatment given and the outcome of the hospital stay. The case definition included both postoperatively colonized and clinically infected cardiac surgery patients, but all cases of the first outbreak episode had clinical signs of infection and received treatment with piperacillin–tazobactam (TZP) or meropenem (MEM). Two of these case patients had a fatal outcome, which was not considered directly related to the postoperative infection by the physician in charge.

**Table 1:** Demographic characteristics, clinical features, treatment and outcome of case patients

Case No.^a^	Age (yrs)	Sex	Type of surgery	Need for revision^b^	Type of infection	Postoperative days to infection^c^	Sample types^d^	Treatment^e^	Outcome^f^
1	42	M	LVAD implantation	No	Pneumonia	28	Sputum, oropharynx, chest tube insertion site, stool, sacral decubitus ulcer	TZP	Discharge
2	55	M	Aortic dissection repair	Yes	Pneumonia	2 (1)	Endotracheal aspirate, sputum, oropharynx, stool	TZP, MEM	Discharge
3	75	F	Aortic valve replacement	Yes	Pneumonia	2 (2)	Sputum, endotracheal aspirate, stool	MEM	Discharge
4	53	M	LVAD implantation, tricuspid annuloplasty	Yes (twice)	CRBSI	12 (7; 6)	Blood, CVC tip, oropharynx, stool	TZP, MEM, CVC removal	Death
5	80	M	CABG	No	Sternal wound infection, mediastinitis	5	Sternal wound fluid, sternal and mediastinal debridement samples	MEM, sternal and mediastinal debridement	Discharge^g^
6	72	F	Mitral valve replacement, tricuspid annuloplasty, maze procedure, PFO closure, LAA exclusion	No	Pneumonia	3	Endotracheal aspirate, oropharynx, BAL fluid, stool	TZP	Discharge
7	78	F	CABG, aortic valve replacement	No	Pneumonia, CRBSI	3	Endotracheal aspirate, blood, CVC tip, AC insertion site, stool	TZP, MEM, CVC removal	Death
8	86	F	Aortic dissection repair	Yes	Pneumonia	2 (1)	Oropharynx, sputum, stool	MEM	Discharge
9	<.1	F	Aortic coarctation repair, pulmonary artery banding	Yes	Tracheo-bronchitis	14 (14)	Oropharynx, endotracheal aspirate	TZP	Discharge
10	.3	M	ASD closure, mitral valvuloplasty	No	Pneumonia	4	Oropharynx, endotracheal aspirate, stool, urine	TZP	Discharge
11	33	M	Heart transplantation	No	Inguinal wound infection^h^	41	Stool, inguinal wound fluid	Local wound care	Discharge
12	<.1	M	Blalock-Taussig shunt placement	No	Pneumonia	7	Oropharynx	TZP, MEM	Discharge
13	83	F	Mitral valve replacement	Yes	Pneumonia, sepsis	2 (1)	Oropharynx, endotracheal aspirate, blood, stool	MEM	Death
14	60	M	CABG, mitral annuloplasty	No	Colonization^i^	–	Oropharynx, endotracheal aspirate	–	Death
15	46	F	Bilateral pulmonary embolectomy	Yes	Pneumonia, UTI	3 (2)	Oropharynx, endotracheal aspirate, stool, urine	TZP, MEM, nitrofurantoin^j^	Discharge
16	62	F	Aortic valve replacement	No	Infectious exacerbation of COPD	2	Sputum, stool	MEM	Discharge
17	64	M	Aortic valve replacement, mitral annuloplasty	No	Pneumonia, sepsis, sternal wound infection	1	Oropharynx, blood, sternal wound fluid	MEM, moxifloxacin, local wound care^k^	Discharge
18	75	M	Aortic valve bioprosthesis replacement, ascending aorta replacement	Yes	Pneumonia	16 (16)^l^	Oropharynx, stool	MEM	Discharge

Abbreviations: *AC* arterial catheter, *ASD* atrial septal defect, *BAL* bronchoalveolar lavage, *CABG* coronary artery bypass grafting, *COPD* chronic obstructive pulmonary disease, *CRBSI* catheter-related bloodstream infection, *CVC* central venous catheter, *ESBL* extended-spectrum β-lactamase, *LAA* left atrial appendage, *LVAD* left ventricular assist device, *MEM* meropenem, *PFO* patent foramen ovale, *TZP* piperacillin–tazobactam, *UTI* urinary tract infection

^a^Cases are numbered in chronological order of occurrence. Dashed lines separate the different outbreak episodes

^b^Postoperative need for urgent reoperation because of bleeding with imminent or manifest pericardial tamponade or because of severe ventricular dysfunction

^c^Number of days between cardiac surgery and collection of the first clinical sample positive for ESBL-producing *E. cloacae* complex. In case of cardiac surgery followed by revision operation(s), values between brackets indicate days between revision operation and infection

^d^All types of clinical and screening specimens from which ESBL-producing *E. cloacae* complex was isolated

^e^When both TZP and MEM are listed, TZP was given first and was later replaced by MEM because of antimicrobial susceptibility testing results or treatment failure

^f^Outcome of the hospital stay. Deaths reflect overall mortality (see text for details on attributable mortality)

^g^Case #5 had to be readmitted after discharge because of relapse of the sternal and mediastinal infection. Retreatment consisted of operative interventions and a prolonged course of high-dose MEM followed by a course of oral trimethoprim–sulfamethoxazole. Full recovery was achieved at the end of the second admission

^h^Case #11 developed postoperative pneumonia caused by an *E. cloacae* complex strain that did not produce ESBL according to double disk synergy testing. An inguinal wound infection following percutaneous femoral vein catheterization occurred later in the postoperative period. An ESBL-producing *E. cloacae* complex strain was cultured from rectal swabs and inguinal wound fluid on postoperative day 19 and day 41, respectively

^i^Case #14 died from low cardiac output and peripheral arterial disease on the third postoperative day, a few hours after the collection of two respiratory samples that turned out to be positive for ESBL-producing *E. cloacae* complex. The available time for developing an overt infection was limited in this patient. We classify this case as being colonized, but a beginning pneumonia cannot be excluded

^j^Case #15 was first treated for pneumonia. TZP was given for 4 days and then switched to MEM for 10 days. The patient developed a UTI caused by ESBL-producing *E. cloacae* complex 2 weeks after MEM had been stopped, for which a course of nitrofurantoin was given

^k^The pneumonia and sepsis in case #17 were treated with MEM. This intravenous therapy was switched to oral moxifloxacin after 10 days, as prolonged treatment was needed for streptococcal endocarditis. Two superficial sternal wounds were primarily treated with local wound care, but the ESBL-producing *E. cloacae* complex in these wounds may also have responded well to moxifloxacin

^l^Case #18 was treated postoperatively with TZP and linezolid for aortic valve bioprosthesis endocarditis. The TZP treatment may have delayed the development of full-blown pneumonia

**Fig. 2:**
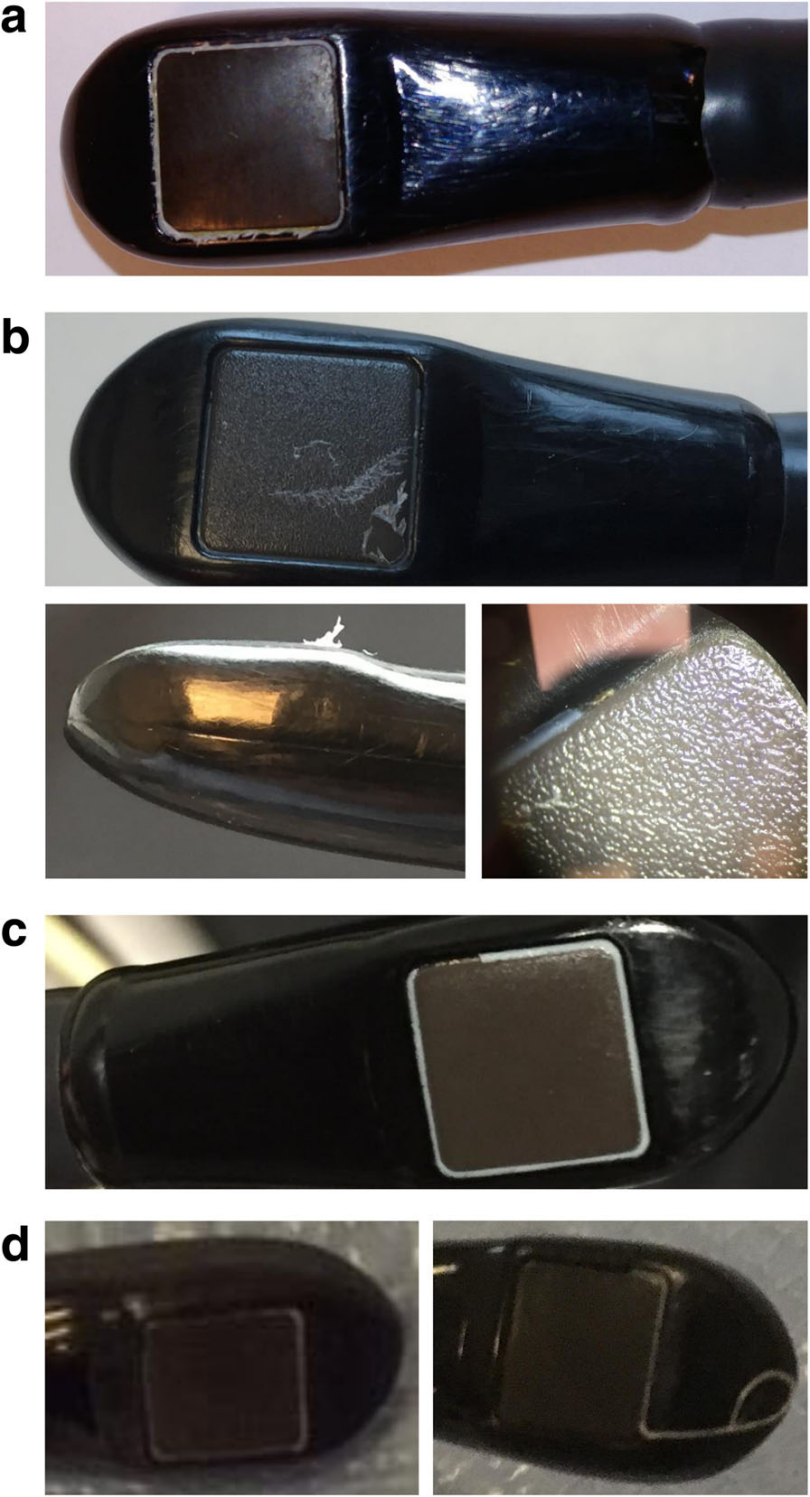
Common pattern of damage of transesophageal echocardiography probes. All affected probes were of the same type (X7-2t transducer; Philips, Amsterdam, The Netherlands). **a** TEE probe of operating room A, beginning of July 2017. The silicone bead around the transducer lens was peeling off and was almost completely missing at one side (the side facing the shaft of the probe). This defect was accompanied by the complete absence of the protective polyethylene film that normally should cover the transducer lens. Shredded polyethylene film fragments can be seen along the remaining parts of the silicone seal. Orange discoloration of some ragged polyethylene film fragments and brown-yellow deposits in the area of the torn-off silicone bead cannot be seen on this picture, but were observed when the TEE probe was examined under a stereoscopic microscope (no pictures available) and were indicative of the presence of cellular debris and organic material. **b** TEE probe of CSICU, mid July 2017. A large part of the silicone bead was missing and the polyethylene film had partially come loose and was ruptured (top). The side view of the probe tip illustrates the detachment and rupture of the polyethylene film (bottom left). Yellow deposits can be seen in the area of the missing silicone bead (bottom right). **c** TEE probe of CICU, end of January 2018. A section of the silicone bead was missing. **d** TEE probe of the cardiology polyclinic, February 2018. The TEE probe appeared intact at the beginning of February 2018 (left), but a new inspection 3 weeks later revealed that a large part of the silicone bead had suddenly come off (right). Abbreviations: *CICU* cardiac intensive care unit, *CSICU* cardiac surgery intensive care unit, *TEE* transesophageal echocardiography

**Fig. 3:**
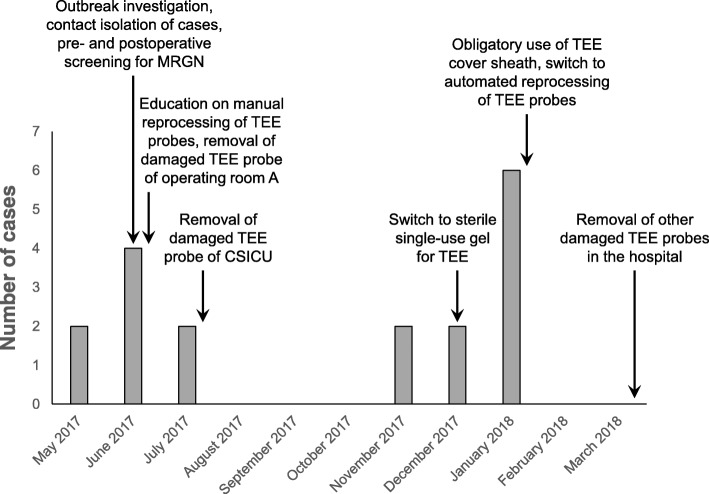
Timeline of cases and key interventions performed to control the outbreak. A more detailed list of all infection control measures taken is shown in Additional file 1: Table S1. Abbreviations: *CSICU* cardiac surgery intensive care unit, *MRGN* multiresistant Gram-negative bacteria, *TEE* transesophageal echocardiography

The presence of ESBL-producing *E. cloacae* complex in respiratory samples in all but one cases and the development of postoperative pneumonia in most cases led us rapidly to consider airway-related procedures as possible source of the outbreak. A major suspected culprit was the performance of TEE examinations in cardiac surgery patients. Typically a TEE probe is inserted through the oropharynx into the esophagus after anesthesia induction and placed in a retrocardiac position for the entire duration of the operation to visualize the structure and function of the heart. Additional TEE examinations may be performed in the postoperative period. Samples were taken from the TEE probes of the cardiac operating rooms and CSICU the end of June 2017, after the first six cases had occurred, but all cultures were negative. Inspection of the TEE probe of operating room A, where five of these six case patients had been operated, showed clear damage to the silicone bead around the transducer lens and the polyethylene film covering the lens (Fig. 2a). It was decided to remove the TEE probe from service. Ten days later two new cases occurred, again with positive respiratory samples. Inspection of the TEE probe of CSICU, which had been used to perform postoperative TEE examinations in both patients, demonstrated a similar pattern of damage (Fig. 2b). Analysis of the incidence rates of ESBL-producing *E. cloacae* complex at other ICUs showed a minor peak at SICU and MICU in June 2017 (two and one new patients, respectively; Fig. 1). Both infected non-cardiac patients at SICU had positive respiratory samples and turned out to have had a TEE examination shortly beforehand, which was very likely performed using the TEE probe of CSICU. This probe was removed from service mid July 2017, after which the first outbreak episode stopped. Figure 3 shows a timeline of cases and key interventions performed to control the outbreak. The entire set of infection control measures is depicted in Additional file 1: Table S1.

A case-control study was performed to identify risk factors and outcome characteristics associated with the development of ESBL-producing *E. cloacae* complex infection following cardiac surgery (Table 2). Compared to 16 control patients, the 8 cases were more likely to have a longer duration of surgery (median, 275 min vs 197 min; *P* = .011) and to have traceable TEE examinations in the postoperative period (OR = 15.40 [95% CI, 1.47–160.97]; *P* = .027). These findings are consistent with the hypothesis of intraoperative and postoperative TEE examinations as source of the outbreak. Cases were more likely than controls to have a preoperative ASA score of 5 (OR = 17.00 [95% CI, 1.59–181.36]; *P* = .007), an emergency operation (OR = 17.00 [95% CI, 1.59–181.36]; *P* = .007) and a postoperative need for urgent revision due to a non-infectious complication (OR = 25.50 [95% CI, 2.36–275.74]; *P* = .001). Bed position E at CSICU was more often occupied by cases than controls (OR = 15.00 [95% CI, 1.29–174.39]; *P* = .028). Cases also had significantly longer length of stay at CSICU compared to controls (median, 15 days vs 3 days; *P* = .006), longer postoperative length of stay (median, 30 days vs 10 days; *P* < .001) and longer total length of stay (median, 34 days vs 12 days; *P* = .001).

**Table 2:** Comparison of preoperative, intraoperative and postoperative characteristics between cases of the first outbreak episode and control patients

Categorical variables	Case patients, *n* (%)	Control patients, *n* (%)	Odds ratio (95% CI)	*P* value
Female sex	4 (50)	2 (13)	7.00 (0.92–53.23)	.129
Smoker^a^	3 (38)	2 (13)	4.20 (0.54–32.96)	.289
Medical history				
Congestive heart failure	4 (50)	5 (31)	2.20 (0.39–12.57)	.412
Hypertension	7 (88)	7 (44)	9.00 (0.89–91.26)	.079
Myocardial infarction	2 (25)	2 (13)	2.33 (0.26–20.66)	.578
Peripheral vascular disease	1 (13)	2 (13)	1.00 (0.08–13.02)	1.000
Cerebrovascular disease	1 (13)	3 (19)	0.62 (0.05–7.12)	1.000
COPD	1 (13)	3 (19)	0.62 (0.05–7.12)	1.000
Diabetes	0 (0)	4 (25)	0.29 (0.03–2.91)	.262
Chronic renal failure	2 (25)	4 (25)	1.00 (0.14–7.10)	1.000
Dialysis	1 (13)	0 (0)	4.25 (0.33–54.07)	.333
Cirrhosis	1 (13)	1 (6)	2.14 (0.12–39.47)	1.000
Solid organ transplantation	0 (0)	1 (6)	0.89 (0.07–11.22)	1.000
Previous cardiac surgery	0 (0)	1 (6)	0.89 (0.07–11.22)	1.000
Preoperative ICUs or wards^b^				
CSICU	2 (25)	1 (6)	5.00 (0.38–66.01)	.249
CICU	1 (13)	5 (31)	0.31 (0.03–3.29)	.621
MICU	0 (0)	1 (6)	0.89 (0.07–11.22)	1.000
SICU	1 (13)	0 (0)	4.25 (0.33–54.07)	.333
Cardiac surgery ward	3 (38)	10 (63)	0.36 (0.06–2.08)	.390
Cardiology ward	2 (25)	3 (19)	1.44 (0.19–11.04)	1.000
Gastroenterology ward	1 (13)	0 (0)	4.25 (0.33–54.07)	.333
General internal medicine ward	0 (0)	1 (6)	0.89 (0.07–11.22)	1.000
Geriatric ward	1 (13)	0 (0)	4.25 (0.33–54.07)	.333
Nephrology ward	1 (13)	0 (0)	4.25 (0.33–54.07)	.333
Preoperative medication^c^				
Antibiotics^d^	2 (25)	4 (25)	1.00 (0.14–7.10)	1.000
Proton pump inhibitors	5 (63)	4 (25)	5.00 (0.81–31.00)	.099
Systemic corticosteroids	1 (13)	1 (6)	2.14 (0.12–39.47)	1.000
Preoperative ASA score of 5	4 (50)	0 (0)	17.00 (1.59–181.36)	.007
Type of cardiac surgery				
Aortic dissection repair	2 (25)	0 (0)	7.29 (0.64–82.62)	.101
Atrial surgery^e^	1 (13)	4 (25)	0.43 (0.04–4.64)	.631
CABG^f^	2 (25)	6 (38)	0.56 (0.08–3.69)	.667
Heart transplantation	0 (0)	1 (6)	0.89 (0.07–11.22)	1.000
LVAD implantation^f^	2 (25)	0 (0)	7.29 (0.64–82.62)	.101
Valve repair or replacement^f^	4 (50)	10 (63)	0.60 (0.11–3.34)	.673
Emergency operation	4 (50)	0 (0)	17.00 (1.59–181.36)	.007
Operating room				
Operating room A	6 (75)	9 (56)	2.33 (0.36–15.30)	.657
Operating room B	1 (13)	7 (44)	0.18 (0.02–1.86)	.189
Operating room C	1 (13)	0 (0)	4.25 (0.33–54.07)	.333
Surgeons				
Surgeon A	4 (50)	8 (50)	1.00 (0.18–5.46)	1.000
Surgeon B	4 (50)	6 (38)	1.67 (0.30–9.27)	.673
Surgeon C	4 (50)	5 (31)	2.20 (0.39–12.57)	.412
Surgeon D	2 (25)	8 (50)	0.33 (0.05–2.18)	.388
Surgeon E	2 (25)	6 (38)	0.56 (0.08–3.69)	.667
Surgeon F	2 (25)	0 (0)	7.29 (0.64–82.62)	.101
Surgeon G	1 (13)	0 (0)	4.25 (0.33–54.07)	.333
Surgeon H	1 (13)	0 (0)	4.25 (0.33–54.07)	.333
Surgeon I	1 (13)	0 (0)	4.25 (0.33–54.07)	.333
Surgeon J	0 (0)	4 (25)	0.29 (0.03–2.91)	.262
Surgeon K	0 (0)	1 (6)	0.89 (0.07–11.22)	1.000
Anesthesiologists				
Anesthesiologist A	5 (63)	7 (44)	2.14 (0.38–12.20)	.667
Anesthesiologist B	3 (38)	6 (38)	1.00 (0.17–5.77)	1.000
Anesthesiologist C	2 (25)	6 (38)	0.56 (0.08–3.69)	.667
Anesthesiologist D	2 (25)	3 (19)	1.44 (0.19–11.04)	1.000
Anesthesiologist E	2 (25)	1 (6)	5.00 (0.38–66.01)	.249
Anesthesiologist F	1 (13)	0 (0)	4.25 (0.33–54.07)	.333
Anesthesiologist G	1 (13)	0 (0)	4.25 (0.33–54.07)	.333
Anesthesiologist H	0 (0)	3 (19)	0.39 (0.04–4.06)	.526
Anesthesiologist I	0 (0)	1 (6)	0.89 (0.07–11.22)	1.000
Anesthesiologist J	0 (0)	1 (6)	0.89 (0.07–11.22)	1.000
Anesthesiologist K	0 (0)	1 (6)	0.89 (0.07–11.22)	1.000
Anesthesiologist L	0 (0)	1 (6)	0.89 (0.07–11.22)	1.000
Surgical antibiotic prophylaxis				
Cefazoline	5 (63)	14 (88)	0.24 (0.03–1.87)	.289
Vancomycin	1 (13)^g^	0 (0)	4.25 (0.33–54.07)	.333
Amoxicillin–clavulanic acid	1 (13)	0 (0)	4.25 (0.33–54.07)	.333
No additional coverage^h^	1 (13)	2 (13)	1.00 (0.08–13.02)	1.000
Sternotomy	7 (88)	12 (75)	2.33 (0.22–25.25)	.631
ECC	8 (100)	16 (100)	-^i^	-^i^
Intraoperative TEE	8 (100)	16 (100)	-^i^	-^i^
Postoperative TEE^j^	7 (88)	5 (31)	15.40 (1.47–160.97)	.027
Need for revision^k^	5 (63)^l^	0 (0)	25.50 (2.36–275.74)	.001
Beds occupied at CSICU^m^				
Bed A	1 (13)	4 (25)	0.43 (0.04–4.64)	.631
Bed B	1 (13)	3 (19)	0.62 (0.05–7.12)	1.000
Bed C	0 (0)	3 (19)	0.39 (0.04–4.06)	.526
Bed D	1 (13)	3 (19)	0.62 (0.05–7.12)	1.000
Bed E	4 (50)	1 (6)	15.00 (1.29–174.39)	.028
Bed F	2 (25)	0 (0)	7.29 (0.64–82.62)	.101
Bed G	2 (25)	1 (6)	5.00 (0.38–66.01)	.249
Bed H	1 (13)	1 (6)	2.14 (0.12–39.47)	1.000
Bed I	1 (13)	2 (13)	1.00 (0.08–13.02)	1.000
Bed J	0 (0)	4 (25)	0.29 (0.03–2.91)	.262
Death^n^	2 (25)	1 (6)	5.00 (0.38–66.01)	.249
Continuous variables	Case patients, median (IQR)		Control patients, median (IQR)	*P* value
Age (yrs)	74 (54–80)		67 (55–77)	.610
BMI (kg/m^2^)	26.5 (24.7–31.0)		26.2 (24.0–28.2)	.492
Preoperative length of stay (days)	2 (0–5)		2 (1–8)	.490
Duration of surgery (minutes)	275 (221–394)		197 (158–223)	.011
Duration of ECC (minutes)	116 (99–356)		106 (79–145)	.198
Length of stay at CSICU (days)	15 (6–31)		3 (1–5)	.006
Postoperative length of stay (days)	30 (26–39)		10 (7–13)	<.001
Total length of stay (days)	34 (26–63)		12 (8–25)	.001

Abbreviations: *ASA* American Society of Anesthesiologists, *BMI* body mass index, *CABG* coronary artery bypass grafting, *CI* confidence interval, *CICU* cardiac intensive care unit, *COPD* chronic obstructive pulmonary disease, *CSICU* cardiac surgery intensive care unit, *ECC* extracorporeal circulation, *ESBL* extended-spectrum β-lactamase, *ICU* intensive care unit, *IQR* interquartile range, *LVAD* left ventricular assist device, *MICU* medical intensive care unit, *SD* standard deviation, *SICU* surgical intensive care unit, *TEE* transesophageal echocardiography

^a^Current smoker. Findings were similar when smoking history was analyzed (data not shown)

^b^Intensive care units or wards where the patient was hospitalized prior to cardiac surgery

^c^Recent medication use (within seven days of cardiac surgery)

^d^Preoperative use of antibiotics for therapeutic purposes (infection). See below for perioperative antibiotic prophylaxis

^e^One or more of the following procedures: left atrial appendage exclusion, maze procedure, patent foramen ovale closure. Atrial surgery was always performed in conjunction with another cardiac surgical procedure

^f^Alone or in combination with another cardiac surgical procedure

^g^Vancomycin was administered prophylactically to one patient who was already on treatment with piperacillin–tazobactam

^h^No additional prophylactic use of antibiotics in patients already on antibiotic treatment

^i^Measures of association were not computed for categorical variables that had a constant value in each of the cases and controls

^j^Traceable TEE examination in the postoperative period. This may be an underestimation of the actual number of patients who had postoperative TEE examinations due to underregistration

^k^Postoperative need for urgent reoperation because of a major non-infectious complication

^l^Four patients required one or more revision operations for bleeding with imminent or manifest pericardial tamponade. Five of these revision operations occurred prior to infection with ESBL-producing *E. cloacae* complex (see Table 1). A fifth patient was reoperated because of postoperative mitral regurgitation with hemodynamic instability. This revision took place shortly after the onset of infection with ESBL-producing *E. cloacae* complex

^m^Beds where the patient stayed at CSICU

^n^Fatal outcome (overall mortality)

### Second outbreak episode

Four months passed without any new cardiac surgery patient having cultures positive for ESBL-producing *E. cloacae* complex. The outbreak, however, unexpectedly recurred mid November 2017, with four new cases being identified over the course of 1 month (Fig. 1). All cases were symptomatic and three of them had an acute respiratory infection with ESBL-producing *E. cloacae* complex in the postoperative period (Table 1). These three cases were infants, whereas all patients of the first outbreak episode were adults, and one infant did not have traceable TEE examinations. This caused uncertainty whether these new cases were related to the first outbreak episode and suggested a multifactorial mode of transmission. All cases recovered after antibiotic treatment and could be discharged from the hospital.

### Third outbreak episode

A new cluster of six cases occurred in January 2018 (Fig. 1). All cases were adults, had undergone TEE examinations and had respiratory samples positive for ESBL-producing *E. cloacae* complex (Table 1). One case died from a non-infectious cause on the third postoperative day and probably did not have the time to develop pneumonia. The other five cases developed clinical infections and were treated with antibiotics. One patient died from ESBL-producing *E. cloacae* complex sepsis secondary to pneumonia.

Similar to the first outbreak episode, there was a minor increase in the incidence rate of ESBL-producing *E. cloacae* complex at SICU and MICU (Fig. 1). Two non-cardiac surgery patients could be identified who had undergone a TEE examination on the same day at MICU and who subsequently developed ESBL-producing *E. cloacae* complex pneumonia. One of these patients also developed a catheter-related bloodstream infection with ESBL-producing *E. cloacae* complex and died a few days later. Inspection of the TEE probe of CICU, which had been used in both patients, showed that a part of the silicone bead around the transducer lens was torn off (Fig. 2c). Measures taken to control the third outbreak episode included, among others, the obligatory use of a protective sheath to cover the TEE probe during cardiac surgery and the decision to switch from manual cleaning and disinfection using chlorine dioxide–generating wipes to automated reprocessing for the TEE probes of the operating rooms and CSICU (Fig. 3, Additional file 1: Table S1). The TEE probe of CICU and the TEE probe of the cardiology polyclinic, which also suffered from detachment of the silicone bead (Fig. 2d), were finally removed from service.

Only one new patient had positive cultures for ESBL-producing *E. cloacae* complex following cardiac surgery in the period from February 2018 to June 2018 (Fig. 1). As this was a single patient without a clear temporal or epidemiological relationship to the cases of January 2018, we did not consider this isolated finding as a new outbreak episode.

### Microbiological and molecular studies

*E. cloacae* complex isolates of the first and second outbreak episode displayed a similar in vitro antibiotic resistance pattern, suggestive of SHV-like ESBL production (clavulanic acid–reversible resistance to third-generation cephalosporins, high-level resistance to trimethoprim–sulfamethoxazole and low-level resistance to ciprofloxacin and gentamicin). Isolates of the third outbreak episode showed a different antibiotic resistance profile (clavulanic acid–reversible resistance to third- and fourth-generation cephalosporins and high-level resistance to trimethoprim–sulfamethoxazole, ciprofloxacin and gentamicin). The high level of resistance to cefotaxime in the latter isolates suggested the production of a CTX-M–like ESBL.

**Fig. 4:**
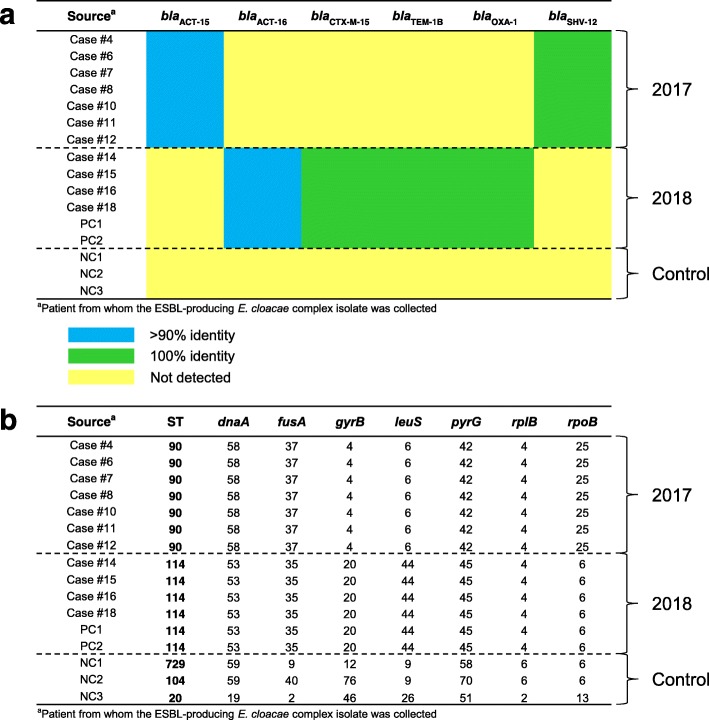
Clonal relatedness of ESBL-producing *E. cloacae* complex isolates. **a** Profile of β-lactam resistance genes. Dashed lines separate the outbreak isolates of 2017 (first and second outbreak episode), the outbreak isolates of 2018 (third outbreak episode, supplemented with two isolates from non-cardiac surgery patients PC1 and PC2) and the negative control isolates (collected in 2017 from non-cardiac surgery patients not involved in the outbreak, denoted as NC1, NC2 and NC3). Blue, presence of a gene sequence in the isolate genome with > 90% identity to a β-lactam resistance gene in the ResFinder database; green, presence of a gene sequence in the isolate genome with 100% identity to a β-lactam resistance gene in the ResFinder database; yellow, resistance gene not detected in the isolate genome. **b** Sequence type as assessed by MLST. For each isolate genome, the best-matching allele at each locus of the MLST scheme was identified and sequence type was then determined by the combined allelic profile. Numbers shown are unique identifiers of alleles and sequence types. Dashed lines separate the outbreak isolates of 2017, the outbreak isolates of 2018 and the negative control isolates. Abbreviations: *ESBL* extended-spectrum β-lactamase, *MLST* multilocus sequence typing, *ST* sequence type

Whole-genome sequencing was performed on isolates that had been collected from four cases of the first outbreak episode (cases 4, 6, 7 and 8), three cases of the second outbreak episode (cases 10, 11 and 12), four cases of the third outbreak episode (cases 14, 15, 16 and 18) and both non-cardiac surgery patients who had a similar TEE-associated infection and disease course in January 2018 as the case patients (PC1 and PC2). Three apparently unrelated ESBL-producing *E. cloacae* complex isolates, collected in October and November 2017 from non-cardiac surgery patients not involved in the outbreak (NC1, NC2 and NC3), were included as negative controls. Alignment of the contigs of the isolate genomes to the collection of β-lactam resistance genes in the ResFinder database [3, 4] revealed an identical profile of β-lactam resistance genes for all isolates of the first and second outbreak episode (*bla*_SHV-12_ with 100% identity and *bla*_ACT-15_ with > 90% identity) and another identical profile for all isolates of the third outbreak episode and both isolates of patients PC1 and PC2 (*bla*_CTX-M-15_, *bla*_TEM-1B_ and *bla*_OXA-1_ with 100% identity and *bla*_ACT-16_ with > 90% identity) (Fig. 4a). In contrast, ResFinder analysis did not detect the presence of any of these β-lactam resistance genes in the strains isolated from patients NC1, NC2 and NC3. Molecular typing using the MLST web tool of the Center for Genomic Epidemiology [5, 6] showed an identical sequence type for all isolates of the first and second outbreak episode (ST90), another common sequence type for all isolates of the third outbreak episode and those from patients PC1 and PC2 (ST114) and three different sequence types for the isolates derived from patients NC1, NC2 and NC3 (ST729, ST104 and ST20, respectively) (Fig. 4b). Collectively, these findings strongly suggest that the first and second outbreak episode were caused by the same strain (SHV-12–producing *E. cloacae* complex ST90 clone), whereas a different strain was at the origin of the third outbreak episode (CTX-M-15–producing *E. cloacae* complex ST114 clone). The presence of an identical molecular pattern in the isolates cultured from patients PC1 and PC2 and the isolates from the case patients of the third outbreak episode adds further evidence to the hypothesis of TEE as mode of transmission, as the performance of a TEE examination was the only identifiable factor common to these two groups of affected patients.

A total of 82 environmental surveillance samples were collected for outbreak investigation, but none of them showed the presence of ESBL-producing *E. cloacae* complex. Periodic sampling of TEE probes, however, identified a transient contamination of the TEE probe of CSICU in February 2018. Four different pathogens were cultured from a swab sample of the TEE probe that had been manually disinfected: ESBL-negative *E. cloacae* complex, *Klebsiella oxytoca*, *Pseudomonas aeruginosa* and *Enterococcus faecalis* (data not shown). A cardiac surgery patient was identified who had undergone a TEE examination with this probe 18 h before the sampling and who was known to have positive cultures for all four microorganisms, with highly similar antibiotic susceptibility patterns, pointing to the very likely point of origin of this contamination event. Careful monitoring of all microbiological laboratory results in the next 2 weeks did not provide evidence of cross-contamination to other patients.

## Discussion

This article describes a protracted outbreak of postoperative infections with ESBL-producing *E. cloacae* complex in cardiac surgery patients, consisting of three episodes and involving two different bacterial strains. Previously published outbreaks of *E. cloacae* infections following cardiac surgery have been linked to surgical complications and cephalosporin prophylaxis [7], liberal use of cephalosporins [8], contamination of an intra-arterial monitoring device [9], contaminated cardioplegia ice [10] and increased use of cefepime and quinolones [11]. There is also one report of a TEE-associated increased incidence of *E. cloacae* isolated from oropharyngeal and sputum samples at a cardiovascular ward, without an accompanying increase in *E. cloacae* pneumonia [12]. We present multiple lines of evidence that point to TEE as the source of the current outbreak, including the clinical presentation of the cases (most typically with respiratory samples positive for ESBL-producing *E. cloacae* complex and development of pneumonia shortly after cardiac surgery), the resolution of the first and third outbreak episode after removal of the damaged TEE probes and institution of other TEE-directed infection control measures, the association of cases with longer surgery duration and traceable postoperative TEE examinations, the occurrence of similar infections in SICU and MICU patients who had undergone TEE and the detection of an identical molecular pattern between the isolates collected from the cases in January 2018 and those from two affected MICU patients, who appeared to have only TEE as common exposure factor. We did not detect ESBL-producing *E. cloacae* complex in any of the samples taken from the TEE probes, but a transient contamination of the TEE probe of CSICU with four other microorganisms was identified. The outbreak seemed to be primarily confined to ESBL-producing *E. cloacae* complex, as is illustrated in Additional file 1: Figure S1 and as is possibly in keeping with the absence of spreading to other patients when the transient contamination event with four other pathogenic bacteria occurred. The reasons for this species and subtype specificity are unclear, but may involve an increased susceptibility of cardiac surgery patients to *Enterobacter* infections [13] and a higher virulence of bacteria carrying antibiotic resistance–encoding plasmids [14]. It is striking that the epidemic returned with a different clone of ESBL-producing *E. cloacae* complex in January 2018. Of note, a highly similar TEE-associated outbreak of ESBL-producing *E. cloacae* complex in cardiac surgery patients occurred in 2015 and 2016 in another university hospital in Belgium [15]. With regard to the interval of 4 months between the first and second outbreak episode, which were caused by the same clone, possible explanations are reintroduction of the strain by a patient or untreated carrier or the presence of an environmental reservoir. Moist surfaces have been described as sources of ESBL-producing *Enterobacteriaceae* in the ICU, providing an opportunity for both outbreak recurrence and continued sporadic isolation of the causative organism in the post-outbreak period [16].

A limitation of this investigation is that we were not able to assign specific TEE probes to cases on a one-by-one basis because of incomplete documentation and underregistration of TEE examinations. Prompt implementation of a system for full traceability was a major demand of the management board of the hospital when the third outbreak episode occurred. It should also be noted that not all cases can simply be attributed to a TEE procedure. For instance, case #12 did not have a traceable TEE examination, suggesting the involvement of an alternative transmission route. Cross-contamination via the hands of healthcare workers is generally considered the main mode of transmission of ESBL-producing bacteria [17] and may perhaps account for a few cases of the reported outbreak.

We cannot be sure whether the TEE-mediated transmission events of ESBL-producing *E. cloacae* complex resulted from human errors in the manual reprocessing and handling of TEE probes or from an intrinsic failure of the cleaning and disinfection procedure. However, a striking observation was the similar pattern of damage of four different TEE probes. It seems highly plausible that irregular surfaces and poorly accessible nooks and crannies resulting from torn-off silicone parts and shredded polyethylene film fragments cannot be adequately disinfected anymore. An important question is what the root cause of this damage is. We have been cleaning and disinfecting the TEE probes manually using chlorine dioxide–generating wipes for several years, according to the manufacturer’s recommendations. The same manual reprocessing method was in use in the other Belgian university hospital that suffered from a TEE-associated outbreak of ESBL-producing *E. cloacae* complex in cardiac surgery patients [15]. Chlorine dioxide is a strong oxidizing agent with broad-spectrum antimicrobial activity. Although the corrosive activity of chlorine dioxide is relatively low compared to other disinfectants [18], there are concerns that it may damage metal and polymer components of endoscopes [19], especially after long-term use [20]. It remains speculative whether the detachment of the silicone bead around the transducer lens and the rupture of the protective polyethylene film were caused by chlorine dioxide–induced corrosion. These defects could, e.g., also reflect a manufacturing problem or material weakness of the TEE probes, which was possibly aggravated by the repetitive mechanical action of wiping. In any case, a common underlying problem is very likely given the occurrence of the same type of damage with four individual devices. Much to our surprise, the compatibility testing report that can be requested from the manufacturer did not alleviate our concerns on a potential damaging effect of chlorine dioxide–generating wipes and could serve as an additional relevant source of information to users of the same equipment. We finally made the decision to implement an automated reprocessing system for the TEE probes of the operating rooms and CSICU, which uses peracetic acid 5% as high-level disinfectant, and to impose the obligatory use of a TEE cover sheath during cardiac surgery.

## Conclusions

In conclusion, this report documents a TEE-associated triphasic outbreak of postoperative infections with two distinct strains of ESBL-producing *E. cloacae* complex in cardiac surgery patients. Appropriate precautions must be taken to prevent and detect damage of TEE probes. The use of safe and effective reprocessing and handling methods is critical in this regard and regular inspection of TEE probe integrity is highly advisable.

## Supplementary information

**Additional file 1: Table S1.** Measures taken to control the outbreak of ESBL-producing *E. cloacae* complex in cardiac surgery patients. **Figure S1.** Monthly incidence rates of selected microorganisms at CSICU.

## Data Availability

All data generated or analysed during this study are included in this published article and its supplementary information file.

## Abbreviations

AC: Arterial catheter
ASA: American Society of Anesthesiologists
ASD: Atrial septal defect
BAL: Bronchoalveolar lavage
BMI: Body mass index
CABG: Coronary artery bypass grafting
CI: Confidence interval
CICU: Cardiac intensive care unit
COPD: Chronic obstructive pulmonary disease
CRBSI: Catheter-related bloodstream infection
CSICU: Cardiac surgery intensive care unit
CVC: Central venous catheter
ECC: Extracorporeal circulation
ESBL: Extended-spectrum β-lactamase
EUCAST: European Committee on Antimicrobial Susceptibility Testing
ICU: Intensive care unit
IQR: Interquartile range
LAA: Left atrial appendage
LVAD: Left ventricular assist device
MALDI-TOF MS: Matrix-assisted laser desorption/ionization time-of-flight mass spectrometry
MEM: Meropenem
MICU: Medical intensive care unit
MLST: Multilocus sequence typing
MRGN: Multiresistant Gram-negative bacteria
OR: Odds ratio
PFO: Patent foramen ovale
SD: Standard deviation
SICU: Surgical intensive care unit
ST: Sequence type
TEE: Transesophageal echocardiography
TZP: Piperacillin–tazobactam
UTI: Urinary tract infection
w/o: Without
