# Supplementary material for: A recurrent and transesophageal echocardiography–associated outbreak of extended-spectrum β-lactamase–producing Enterobacter cloacae complex in cardiac surgery patients
Source: Antimicrob Resist Infect Control. 2019 Sep 18;8:152. doi: 10.1186/s13756-019-0605-4 (PMC6751596; doi:10.1186/s13756-019-0605-4)

**Table S1.** Measures taken to control the outbreak of ESBL-producing *E. cloacae* complex in cardiac surgery patients

| Establishment of a multidisciplinary outbreak control team | |
| --- | --- |
| Daily monitoring and regular outbreak meetings | |
| Epidemiological and environmental investigation^a^ | |
| Contact isolation of cases | |
| Preoperative screening of all cardiac surgery patients for multiresistant Gram-negative bacteria | |
| Weekly postoperative screening of all cardiac surgery patients for multiresistant Gram-negative bacteria | |
| Use of a disposable single-use sheath to cover the TEE probe during cardiac surgery^b^ | |
| Replacement of non-sterile multi-dose ultrasound transmission gel by sterile single-use gel for TEE procedures^c^ | |
| Establishment of a written hospital-wide protocol for manual cleaning and disinfection of TEE probes | |
| Attempts to improve the manual reprocessing method using chlorine dioxide as high-level disinfectant (Tristel Trio Wipes System; Tristel, Snailwell, UK) by organizing training sessions and hanging up posters^d,e^ | |
| Switch from foam covers that were reused to disposable single-use foam covers for protecting the TEE probe tip after reprocessing | |
| Switch from storage of the TEE probe of CSICU in a non-disinfectable transportation case to suspension in a cupboard after reprocessing^f^ | |
| Replacement of TEE probes^g^ | |
| Extra round of manual cleaning and disinfection of the TEE probes of the operating rooms at the start of each day^h^ | |
| Switch from manual reprocessing to automated reprocessing of the TEE probes of the operating rooms and CSICU (Soluscope Serie TEE reprocessing system, using peracetic acid 5% as high-level disinfectant; Soluscope, Aubagne, France)^h^ | |
| Storage of reprocessed TEE probes in disinfected trays that are sealed with a plastic sheath^h^ | |
| Implementation of tracking procedures for all TEE probes^h^ | |
| On-site visits of the outbreak support team of the Flemish Agency for Care and Health to assist with infection control^h^ | |
| Reporting of the detachment of the silicone seal around the transducer lens of four TEE probes of the same type to the vigilance unit of the Belgian Federal Agency of Medicines and Health Products^h^ | |
| Abbreviations: *CICU* cardiac intensive care unit, *CSICU* cardiac surgery intensive care unit, *ESBL* extended-spectrum β-lactamase, *TEE* transesophageal echocardiography  ^a^See Methods section for details  ^b^There was some resistance to the use of a protective sheath because of image quality concerns. The use of a sheath was recommended from the first outbreak episode and was made obligatory at the third outbreak episode  ^c^Switching to sterile single-use ultrasound transmission gel was already advised at the first outbreak episode, but was only implemented as of the second outbreak episode  ^d^This measure was taken as observation of the manual cleaning and disinfection procedure by the hospital infection control team had shown that the contact time with chlorine dioxide did not always meet the recommended minimum of 30 seconds  ^e^Measure applies only to the first outbreak episode  ^f^Measure applies to the first and second outbreak episode  ^g^The TEE probes of operating room A and CSICU were replaced at the first outbreak episode. The TEE probes of CICU and the cardiology polyclinic were replaced after the occurrence of the third outbreak episode  ^h^Measure applies only to the third outbreak episode |  |

**Figure S1.** Monthly incidence rates of selected microorganisms at CSICU. **a** Incidence rates of ESBL-negative *E. cloacae* complex and *E. aerogenes*. **b** Incidence rates of *P. aeruginosa*, *K. oxytoca* and *E. faecalis*, which are, together with ESBL-negative *E. cloacae* complex, the microorganisms that have been isolated from the transiently contaminated TEE probe of CSICU in February 2018. Abbreviations: *CSICU* cardiac surgery intensive care unit, *ESBL* extended-spectrum β-lactamase, *TEE* transesophageal echocardiography
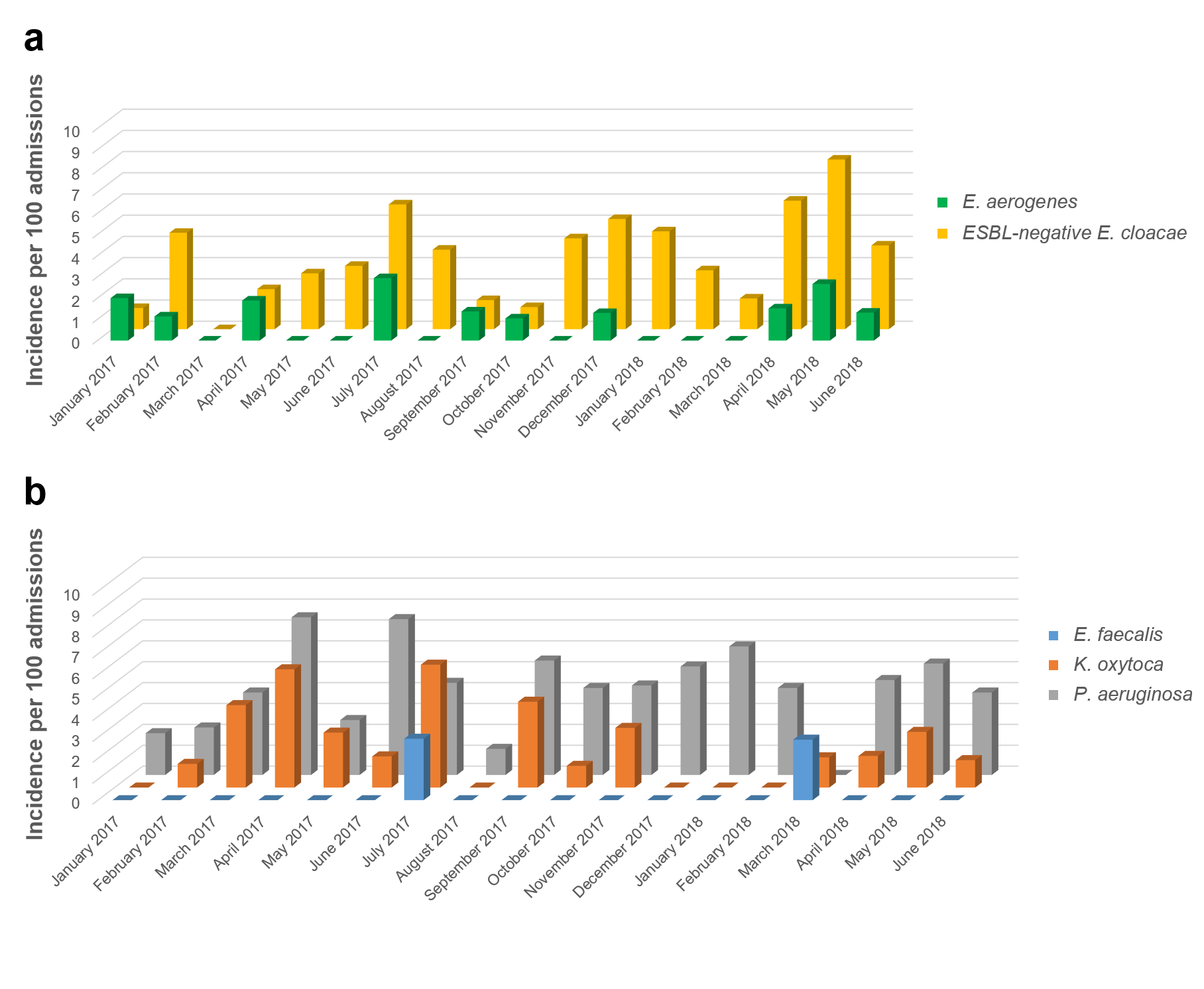

Supplement: Supplementary file 1 — Additional file 1: Table S1. Measures taken to control the outbreak of ESBL-producing E. cloacae complex in cardiac surgery patients. Figure S1. Monthly incidence rates of selected microorganisms at CSICU. [file 13756_2019_605_MOESM1_ESM.docx]
